# Lactoferrin and zinc sulfate supplementation alleviate cisplatin-induced taste disorder: underlying mechanisms via taste and immune modulation

**DOI:** 10.1038/s41538-026-01096-9

**Published:** 2026-08-27

**Authors:** Aili Wang, Wenwen Yu, Xinyi Li, Changwei Luo, Xuanbo Liu, Liang Zou

**Affiliations:** 1 College of Food and Biological Engineering, Key Laboratory of Coarse Cereal Processing (Ministry of Agriculture and Rural Affairs), Chengdu University, Chengdu, China; 2 Department of Food Science and Technology, Virginia Polytechnic Institute and State University (Virginia Tech), Blacksburg, VA USA

**Keywords:** Biochemistry, Diseases

## Abstract

Taste disorder is a common chemotherapy-associated consequence that markedly suppresses the appetite of patients and impacts their quality of life. This study investigated the mechanisms of dietary supplementation of lactoferrin (LF), alone or combined with ZnSO₄, in alleviating cisplatin-induced metallic taste disorder using a mouse model. Two-bottle preference (TBP) test demonstrated that both LF and LF+ZnSO₄ supplementation significantly decreased the metallic taste preference ratio from 82.5 ± 9.4% to 24 ± 5.4% and 20 ± 4.0%, respectively. Transcriptomic analysis revealed that LF upregulated (*P* < 0.05) genes associated with metallic taste receptor, signal transduction, and immune responses, while downregulating genes associated with Fe^2+^-induced lipid oxidation pathway, which might contribute to taste recovery and mucosal defense. ZnSO₄ in combination with LF further elevated the expression of genes involved in metallic taste signaling and intracellular mediators. Co-application of LF and ZnSO₄ was associated with additional changes in the expression of immune-related genes in the oral epithelium; however, this transcriptional difference was not accompanied by a further improvement in taste behavior. The results of this study highlight LF and ZnSO₄ supplementation as promising dietary strategies to alleviate chemotherapy-induced metallic taste disorder and shed light on the underlying mechanisms.

## Introduction

Taste disorder refers to a pathological alteration in gustatory function, including reduced sensitivity (hypogeusia), complete loss (ageusia), or distorted taste perception (dysgeusia)^1^. Such disorders are frequently observed in cancer patients receiving chemotherapy, with over 70% of this population reporting a persistent metallic taste with or without food intake^2^. Metallic taste disorder may persist several hours, weeks, or even months, regardless of the type of chemotherapeutic agent^1,3^. Correspondingly, patients always suffer weight loss, diminished nutrition, and depression due to poor appetite, which largely impacts the quality of life^2^.

Although metallic taste disorder has gained growing attention in recent years, effective therapeutic strategies for its prevention or treatment are still lacking^3^. Lactoferrin (LF) is a naturally occurring iron-binding glycoprotein with a broad range of biological activities, including antimicrobial, antiviral, anticancer, antioxidant, and immunomodulatory functions^4^. In our previous study, lactoferrin (LF) supplementation effectively alleviated the metallic taste symptoms in chemotherapy patients in a small-scale clinical trial^5^. However, the underlying mechanisms, particularly the gene-level molecular mechanisms, remain poorly understood and require further investigation.

Zinc is a component of the salivary enzyme carbonic anhydrase VI, which serves as a growth factor to stimulate sensory stem cell growth and differentiation^6^. Taste disorder was found to correlate with decreased serum zinc level in chemotherapy patients^7,8^. Zinc supplementation has shown its potential as a therapeutic agent for taste dysfunctions in recent studies; however, its molecular mechanisms in improving taste dysfunction are still unclear^9,10^. Moreover, the combination of zinc with other therapeutic approaches (such as Δ-9-tetrahydrocannabinoid) proved particularly promising^9^. Thus, it is meaningful to explore whether the co-application of zinc and LF would further enhance the effects in alleviating taste disorder and investigate the underlying mechanisms.

The aim of this study is to investigate the molecular mechanisms by application of LF, alone or in combination with zinc, in improving chemotherapy-induced metallic taste disorder using a mouse model. The effects of LF and LF+ZnSO_4_ on improving taste disorder will be validated through a two-bottle preference test in mice. At the same time, changes in gene transcription levels before and after LF or LF+ZnSO_4_ treatments will be analyzed. Elucidating these mechanisms is essential for developing targeted therapeutic strategies to improve the nutritional status and quality of life in chemotherapy patients, ultimately guiding clinical recommendations for effective management of taste disorder.

## Results

### Effects of cisplatin on taste function of mice (Experiment 1)

**Fig. 1:**
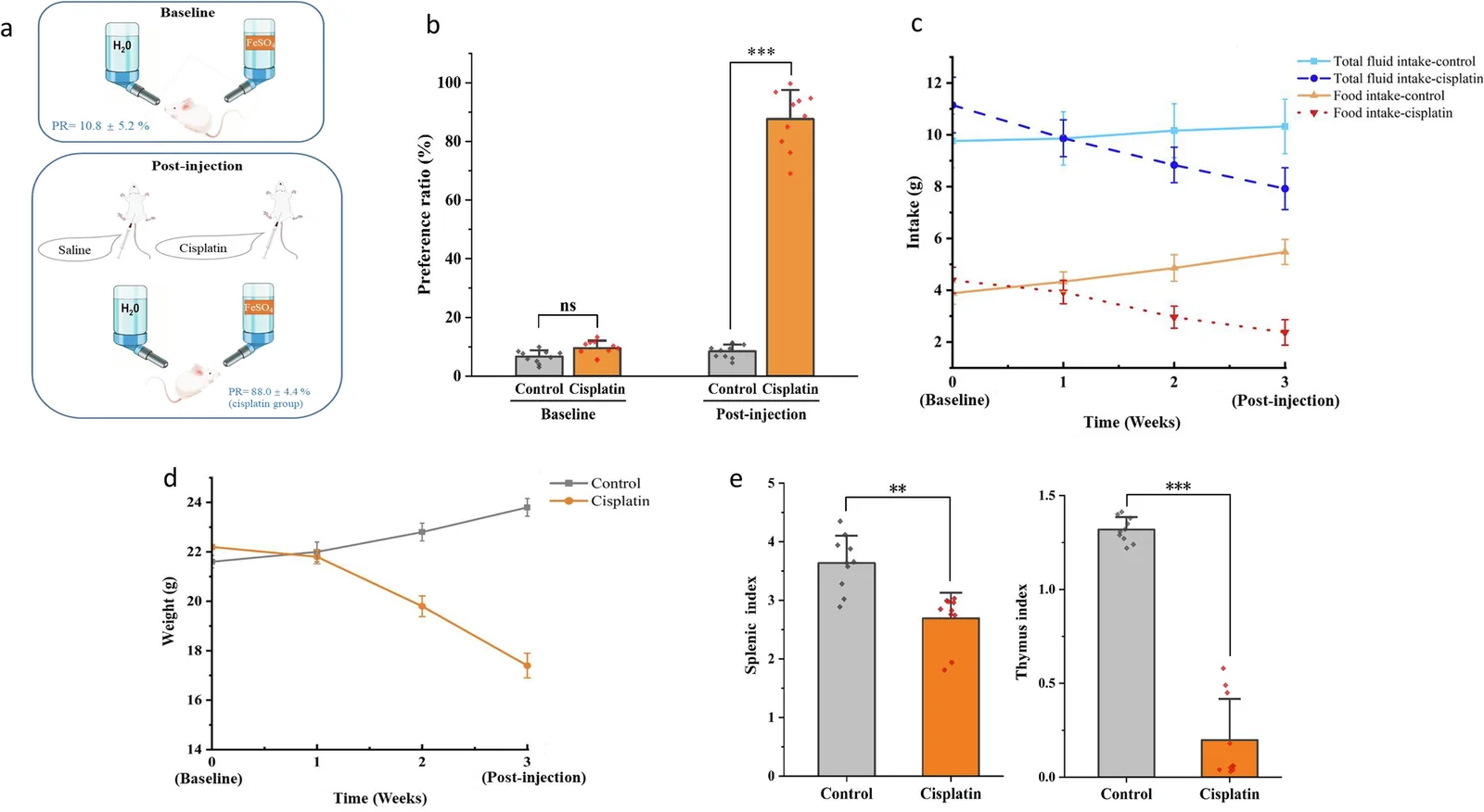
Cisplatin-induced changes in metallic taste preference and physiological parameters in mice. Schematic diagram of the TBP test (**a**), metallic taste preference ratios (**b**), total fluid and food intake (**c**), and body weight (**d**) of mice in control and cisplatin groups at baseline and post-injection stage. Thymus and spleen indices of mice in control and cisplatin group at post-injection stage (**e**). All data are presented as means ± standard deviation. Statistical significance is determined by Student’s *t*-test and is represented by asterisks: ^***^P < 0.001, ^**^P < 0.01, ^*^P < 0.05.

We first screened the female mice that were sensitive to the metallic taste using the TBP test (deionized water, 3.2 mg/L FeSO₄ solution). The rationale and data supporting the choose of female mice in this experiment were provided in the supplementary materials (Fig. S1). The metallic taste PR of selected mice (*n* = 20) was 10.8 ± 5.2%, which was the initial stage of the experiment (baseline, Fig. 1a). Then the mice were divided into two groups for saline and cisplatin injection, respectively. As shown in Fig. 1b, there was no significant difference (*P* > 0.05) in metallic taste PR between the control and cisplatin groups at baseline. However, after 3 weeks of injection, mice of cisplatin group exhibited significantly higher (*P* < 0.05) metallic taste PR compared with the mice in the control group. Cisplatin-treated mice shifted from avoidance toward an active preference for the FeSO₄ solution. Although Ren et al.^11^ reported that cisplatin broadly blunts taste-nerve and behavioral responses across multiple taste modalities, a generalized reduction in taste sensitivity would be expected to drive intake toward indifference (PR ~ 50%) rather than toward an active preference (PR ~ 82%). This behavioral change is unlikely to be explained by general taste impairment alone and is more consistent with an alteration in metallic taste processing.

Both total fluid and food intake of the cisplatin group were significantly lower (*P* < 0.05) than those of the control group by the end of the injection period, which indicated that cisplatin suppressed overall consumption (Fig. 1c). Accordingly, the body weight of mice in cisplatin group was significantly lower (*P* < 0.05) than that of the control group (Fig. 1d). Although total fluid intake declined in the cisplatin group, the PR increased markedly (from ~11% to ~82%). This result indicated that the shift in preference was driven by a redistribution between the two bottles, in which FeSO₄ increased while water intake decreased. Besides, the thymus and spleen indices that were indicators of immune function were also significantly decreased (*P* < 0.01) in mice of the cisplatin group, suggesting the immune function of mice might be damaged by cisplatin (Fig. 1e). Previous studies have reported that immune responses impaired regeneration of both olfactory sensory neurons and taste receptor cells^12^, which might result in metallic taste disorder in this study.

### Effects of cisplatin on gene expression in mice tongue (Experiment 1)

**Fig. 2:**
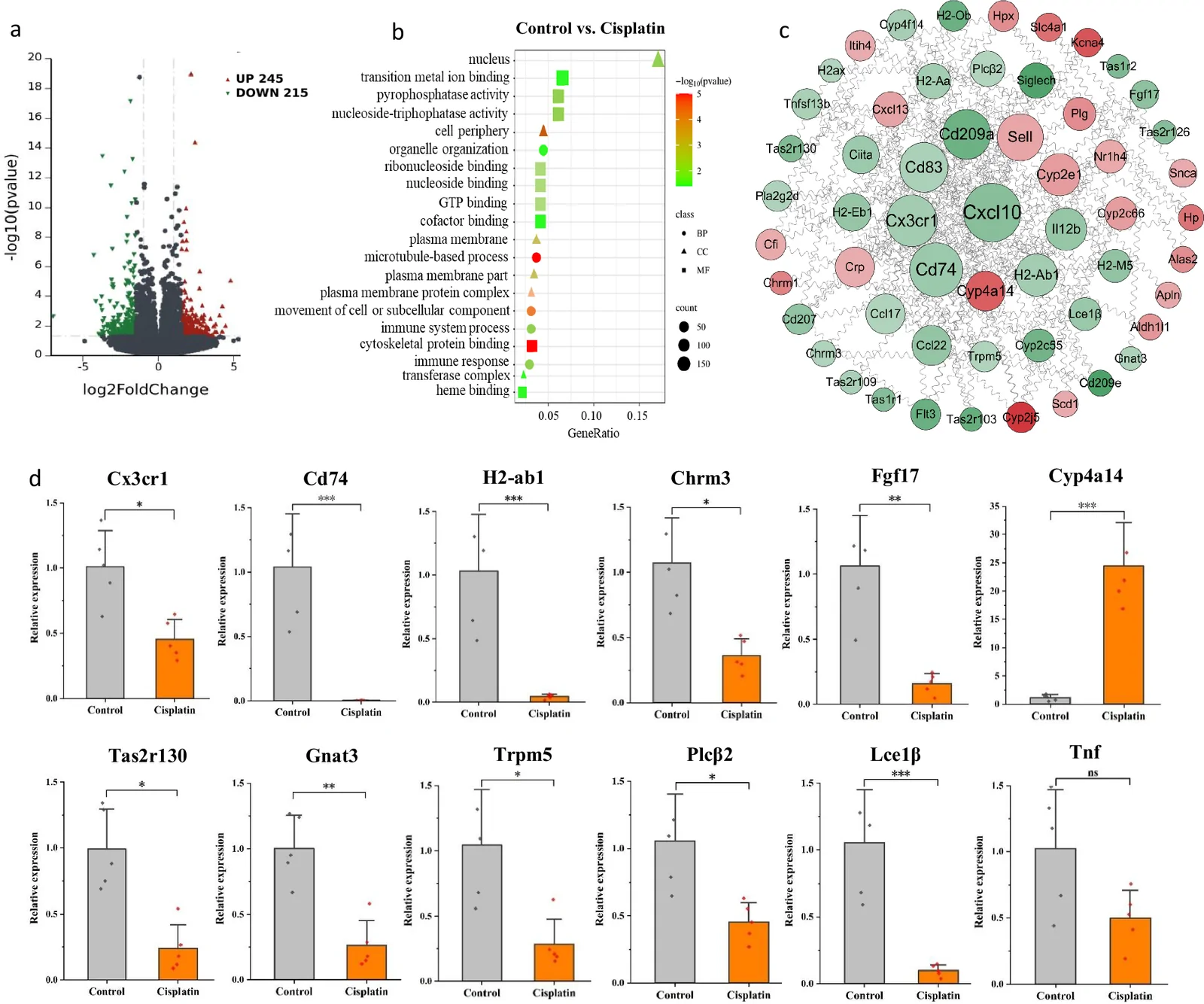
Cisplatin-induced transcriptional alterations in mouse tongue. RNA-Seq analysis of transcriptional alterations in mouse tongue between the control and cisplatin groups after 3 weeks of cisplatin injection, including (**a**) volcano plot of differentially expressed genes (DEGs), (**b**) bubble plot of GO enrichment analysis showing the top 30 significantly (*P* < 0.05) enriched terms across biological process (BP), cellular component (CC), and molecular function (MF), (**c**) protein-protein interaction (PPI) network of DEGs, in which red nodes indicate upregulated genes and green nodes indicate downregulated genes, deeper color intensity reflects greater fold change and larger node size corresponds to higher degree values, and (**d**) expression levels of key genes associated with metallic taste and immune response validated by quantitative PCR. ns, not significant, ^*^P < 0.05, ^**^P < 0.01, ^***^P < 0.001, by Student’s *t*-test.

To investigate the mechanism of cisplatin on the metallic taste disorder in mice, we analyzed the differentially expressed genes (DEGs) of mice tongue between the cisplatin and control groups. The volcano plot displayed a total of 460 DEGs (*P* < 0.05 and |log2FC| ≥ 1) in cisplatin group compared to the control, including 245 upregulated genes and 215 downregulated genes (Fig. 2a). Gene Ontology (GO) enrichment analysis demonstrated that the DEGs were primarily associated with functions such as transition metal ion binding (molecular function, MF), cytokine receptor binding (MF), heme binding (MF), immune system processes (biological process, BP), immune response (BP), and plasma membrane protein (cellular component, CC) (Fig. 2b).

PPI network analysis based on DEGs across experimental groups was performed and shown in Fig. 2c. Hub genes including Cxcl10, Cd74, Cx3cr1, Cd83, Cd209a, Sell, H2-Ab1, Il12b, Cyp2e1, Cyp4a14 were identified according to node degree values, with larger nodes indicating higher connectivity. Quantitative PCR analysis was performed to validate the expression of critical genes and their expression trends were consistent with the RNA-seq results (Fig. 2d).

### Effects of lactoferrin and ZnSO_4_ supplementation on taste behavior of mice injected with cisplatin (Experiment 2)

**Fig. 3:**
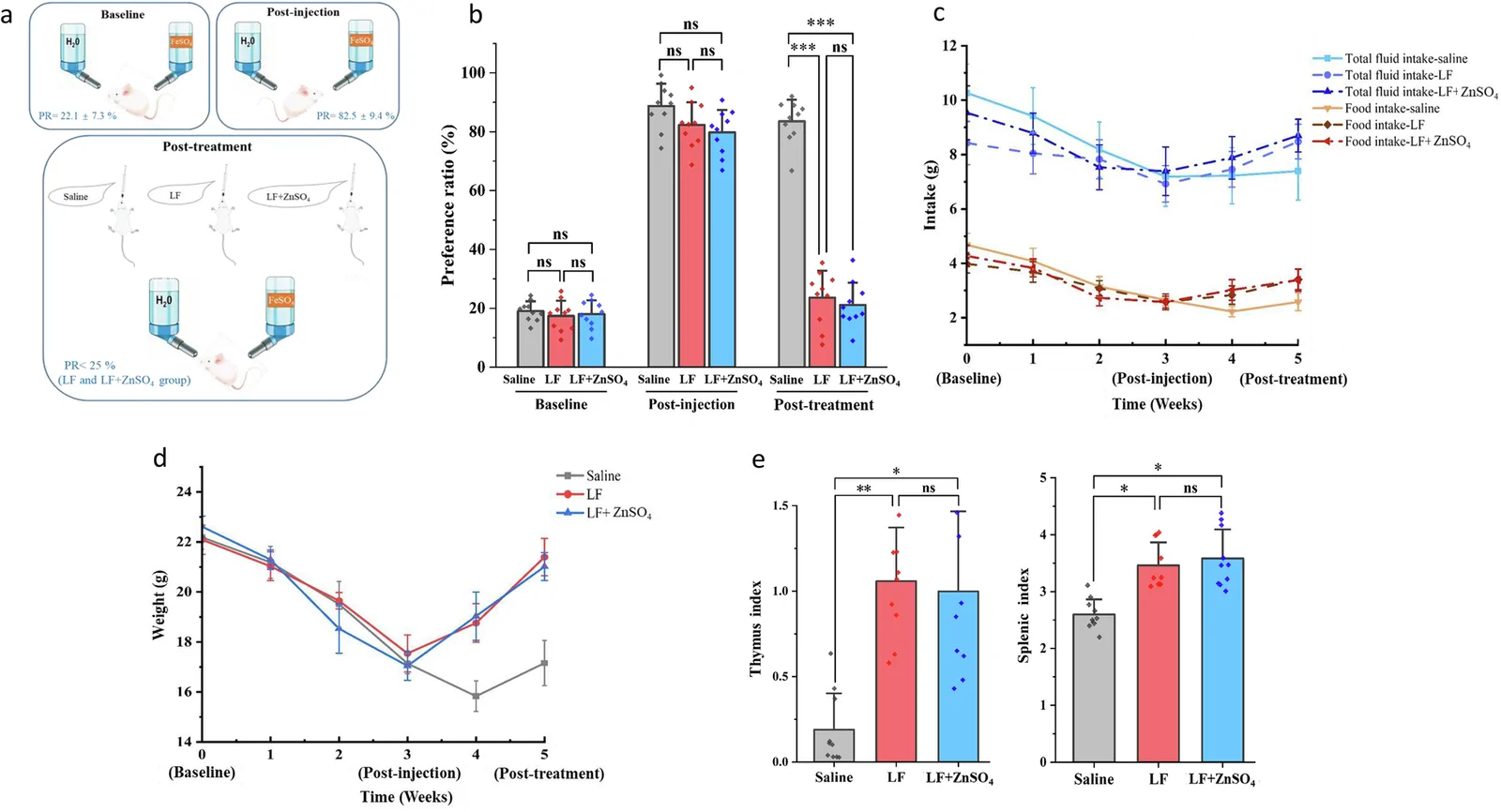
Eff ects of lactoferrin and zinc supplementation on metallic taste preference and physiological parameters in cisplatin-treated mice. Schematic diagram of the TBP test (**a**), metallic taste preference ratios (**b**), total fluid and food intake (**c**), and body weight (**d**) of mice in the saline, LF, and LF+ZnSO_4_ groups at baseline, post-injection, and post-treatment stages. Thymus and spleen indices of mice in the saline, LF, and LF+ZnSO_4_ groups at post-treatment stage (**e**). All data are presented as means ± standard deviation. Asterisks indicate statistical significance determined by one-way ANOVA with Dunnett’s multiple-comparisons test: ^***^P < 0.001, ^**^P < 0.01, ^*^P < 0.05.

At baseline, the metallic taste PR of mice (*n* = 40) was 22.1 ± 7.3% (Fig. 3a). After 3 weeks of injection, two mice died during cisplatin administration and the remaining 38 mice were evaluated. The 30 mice exhibiting the highest metallic taste PR were selected for subsequent experiments and the average PR was 82.5 ± 9.4% (post-injection stage, week 3). The mice (*n* = 30) were then randomly divided into three groups (saline, LF, LF+ZnSO₄) for oral gavage. As shown in Fig. 3b, the metallic taste PR of both LF and LF + ZnSO₄ groups significantly decreased after 14 days of oral gavage (*P* < 0.001), which were close to the baseline values. In contrast, the PR of the saline (control) group did not significantly change (*P* > 0.05) after 14-day oral gavage. Our results indicated that the taste function of mice was unable to self-recover within 14 days after cisplatin injection at 7.0 mg/kg/week for 3 weeks. Although Ren et al.^11^ reported that the expression levels of most genes related to taste cells recovered at Day 14 post-cisplatin injection (7.5 mg/kg, single dose at day 0), their injection dose was much lower than our study and whether the taste function of mice recovered to normal has not been evaluated. Furthermore, in mouse models of cyclophosphamide-induced (chemotherapeutic agent) taste loss, most mice gradually recover their taste function within 16 to 20 days^13,14^. Although selecting the most severely affected animals raised the possibility of regression-to-the-mean bias, this was mitigated by the behavior of the saline control group. Because saline-treated animals were randomly assigned from the identical high-PR pool yet exhibited no spontaneous recovery, the improvements observed in LF and LF+ZnSO₄ groups were more plausibly reflective of a true therapeutic response.

Changes in total fluid intake, food intake, and body weight of mice were consistent with the alterations in taste function. At the post-injection stage (week 3), fluid intake, food intake, and body weight all reached their lowest values, consistent with the reduced appetite accompanying taste disorder (Fig. 3c, d). Administration of ZnSO₄ and/or LF improved the taste disorder and appetite of mice; both intake measures and body weight recovered progressively over the following 2 weeks, coinciding with the alleviation of metallic taste preference. In contrast, mice in the saline group continued to lose weight after the cessation of cisplatin injection, and although a modest recovery was observed from week 5, fluid intake, food intake, and body weight in the saline group remained significantly lower (*P* < 0.05) than those in the LF and LF+ZnSO₄ groups at the post-treatment stage (Fig. 3c, d). The thymus and spleen indices of both LF and LF + ZnSO₄ groups were significantly higher (*P* < 0.05) than those of the saline group at the post-treatment stage (Fig. 3e). Our results suggested that LF and ZnSO₄ might alleviate cisplatin-induced damage to immune organs in mice.

### Effects of lactoferrin on gene expression in mice tongue (Experiment 2)

**Fig. 4:**
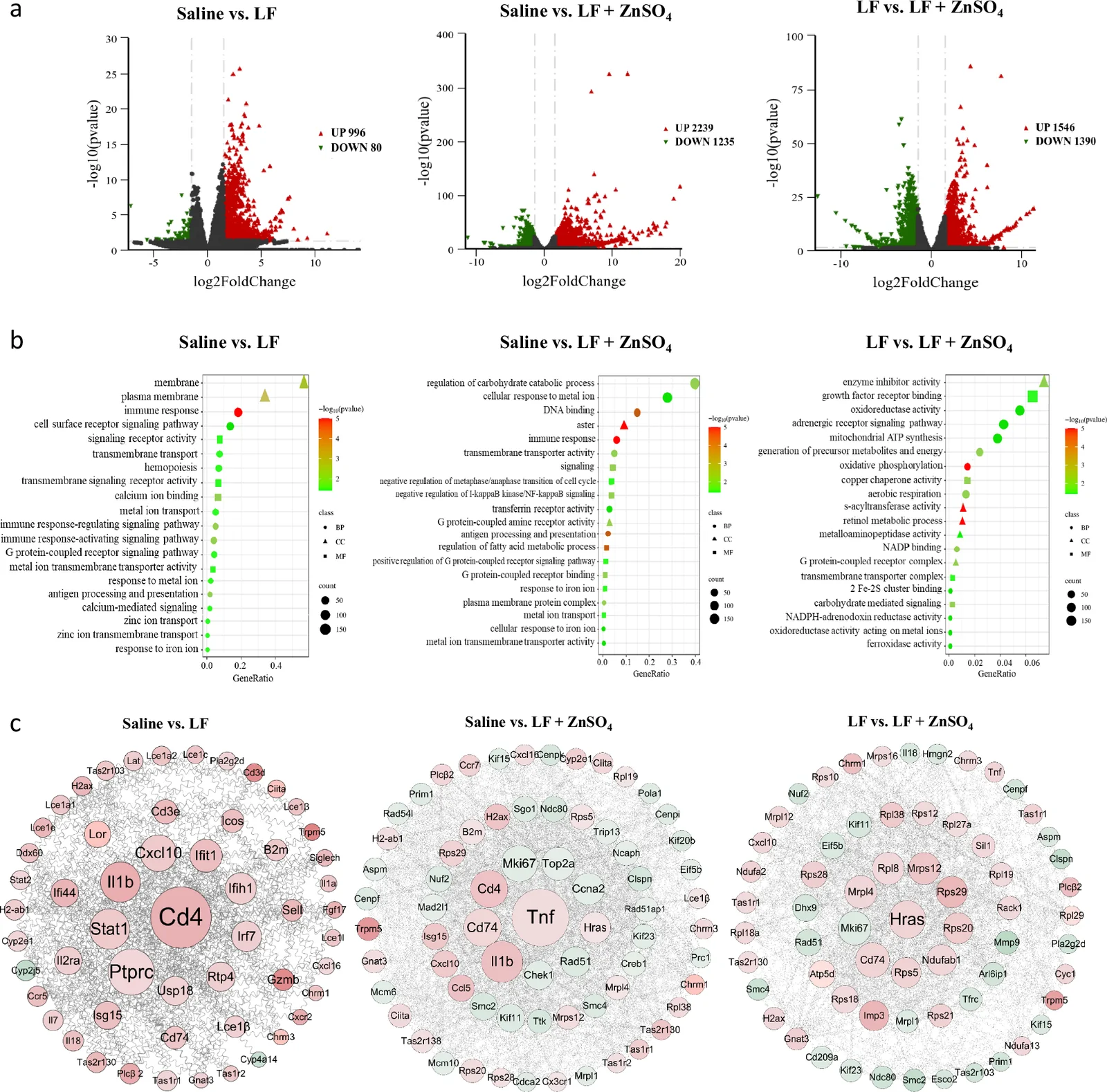

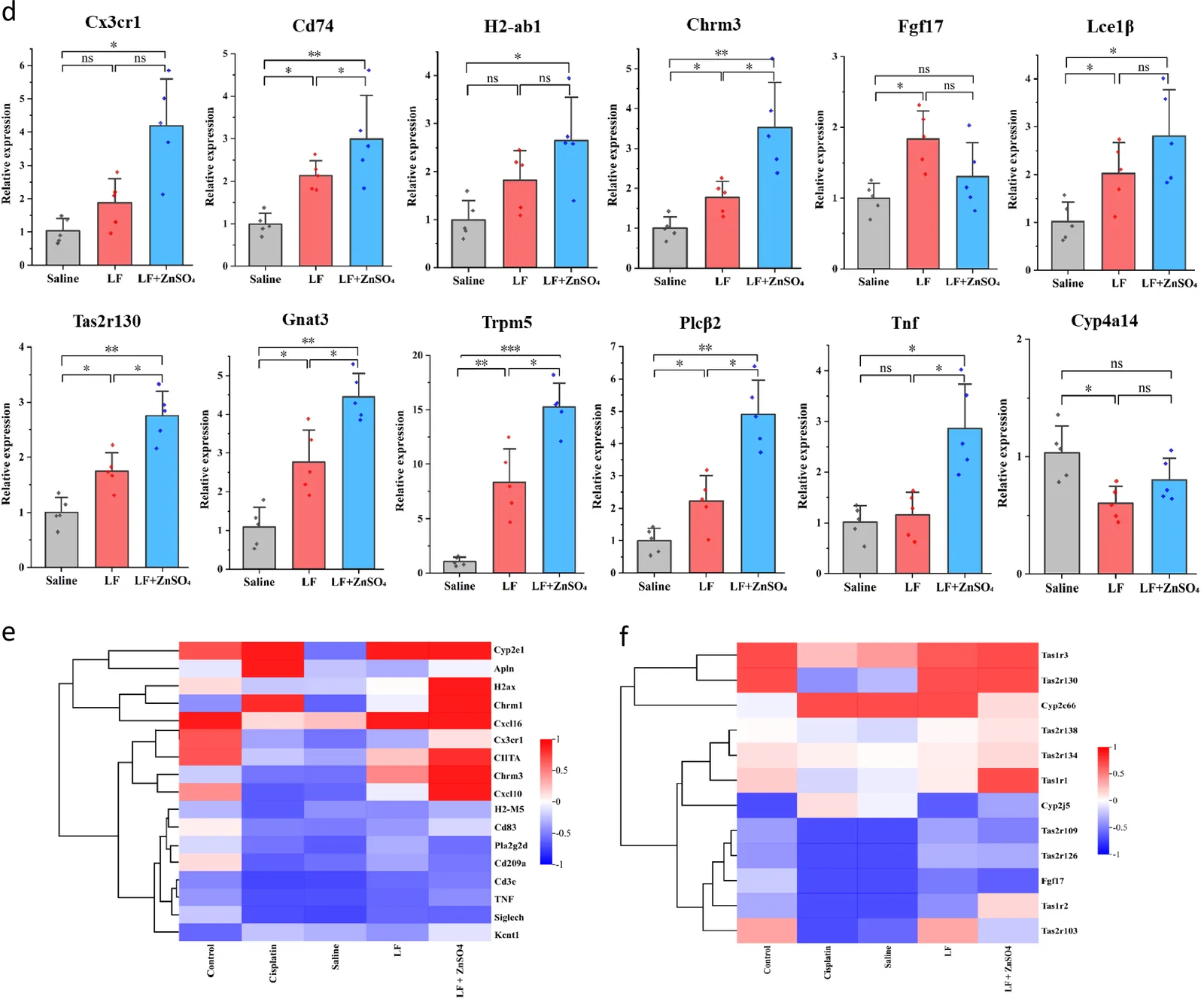
Eff ects of lactoferrin and zinc supplementation on tongue transcriptional profi les in cisplatin-treated mice. RNA-Seq analysis of transcriptional alterations in mouse tongue among saline, LF, and LF+ZnSO_4_ groups after 2 weeks of oral gavage, including (**a**) volcano plot of differentially expressed genes (DEGs), (**b**) bubble plot of GO enrichment analysis showing the top 30 significantly (*P* < 0.05) enriched terms across biological process (BP), cellular component (CC), and molecular function (MF), (**c**) protein-protein interaction (PPI) network of DEGs, in which red nodes indicate upregulated genes and green nodes indicate downregulated genes, deeper color intensity reflects greater fold change and larger node size corresponds to higher degree values, and (**d**) expression levels of key genes associated with metallic taste and immune response validated by quantitative PCR; heatmap showing genes associated with inflammatory responses and intracellular signaling (**e**) as well as taste receptors and functions (**f**) across different treatments. ns, not significant, ^*^P < 0.05, ^**^P < 0.01, ^***^P < 0.001, by one-way ANOVA with Dunnett’s multiple-comparisons test.

The differentially expressed genes (DEGs) of mice tongue between the LF and saline groups were shown in a volcano plot (Fig. 4a). Compared to the saline group (control), there were 996 upregulated genes and 80 downregulated genes (*P* < 0.05 and |log2FC| ≥ 1) in LF group. GO enrichment analysis revealed that DEGs in the LF group were primarily enriched in terms such as, transmembrane signaling receptor activity (MF), signaling receptor activity (MF), immune response (BP), metal ion transport (BP), response to iron ion (BP), antigen processing and presentation (BP), and the G protein-coupled receptor signaling pathway (BP), indicating modulation of immunological signaling (Fig. 4b). PPI network disclosed the top 10 hub genes in LF treatment when compared to the control, including Cd4, Cxcl10, Ifit1, Ifih1, Irf7, Rtp4, Usp18, Ptprc, Stat1, and Il1b (Fig. 4c).

### Effects of co-application of lactoferrin and ZnSO_4_ on gene expression in mice tongue

As shown in volcano plot (Fig. 4a), co-application of LF and ZnSO_4_ significantly increased the expression of 2239 genes and decreased the expression of 1235 genes (*P* < 0.05 and |log2FC| ≥ 1) compared to saline group, while increasing the expression of 1546 genes and decreasing the expression of 1390 genes (*P* < 0.05 and |log2FC| ≥ 1) compared to LF group.

Compared with the saline group, GO enrichment analysis showed that DEGs in LF + ZnSO₄ group were enriched in terms including signaling and G protein-coupled receptor binding (MF), metal ion transport (MF), response to iron ion (MF), cellular response to metal ions (BP), immune response (BP), and transferrin receptor activity (BP), suggesting enhanced immunomodulatory and metal-responsive pathways (Fig. 4b). Comparative analysis between the LF and LF + ZnSO₄ groups revealed that DEGs were enriched in functions such as growth factor receptor binding (MF), generation of precursor metabolites and energy (BP), oxidoreductase activity acting on metal ions (BP), [2Fe–2S] cluster binding (BP), and G protein-coupled receptor complex (CC), suggesting ZnSO₄ may modulate metal-dependent metabolic and mitochondrial redox pathways (Fig. 4b).

PPI network disclosed the top 10 hub genes in LF + ZnSO_4_ treatment when compared to the control, including Tnf, Mki67, Top2a, Ccna2, Hras, Rad51, Chek1, Il1b, Cd74, and Cd4 (Fig. 4c). For visualization, heatmaps (Fig. 4e, f) display representative differentially expressed genes (DEGs) associated with inflammatory responses, intracellular signaling, and taste functions according to GO terms and published literature. As shown in Fig. 4e, genes associated with inflammatory responses (Cxcl10, Cxcl16, Cx3cr1), intracellular signaling (Chrm3, Kcnt1), and taste cell growth (H2ax) were significantly downregulated after cisplatin injection, and their expression levels showed little recovery after 14 days in the absence of LF or LF+ZnSO₄ intervention. LF supplementation partially recovered the expression of these genes, while co-application of LF and ZnSO₄ was associated with a larger increase in their transcript levels. A similar trend was observed for genes associated with taste receptor and signaling transduction, which were broadly downregulated following cisplatin exposure (Fig. 4f). Although partial recovery of these genes was observed in the saline group after 14 days, their levels remained significantly lower than those of the control group. In contrast, both LF and LF+ZnSO₄ treatments significantly accelerated this recovery process. Compared to LF group, the LF+ZnSO₄ treatment further enhanced the expression of taste receptors including Tas2r138, Tas2r134, Tas1r1, Cyp2j5, and Tas1r2.

## Discussion

In the current study, the metallic-taste receptor Tas2r130 and its downstream signaling pathways (Gnat3, Plcβ2, and Trpm5) were significantly downregulated (*P* < 0.05) in the cisplatin group. Among the 25 human bitter taste receptors, only Tas2r7 (Tas2r130 in mice) responds to divalent and trivalent metal ions and activates downstream taste signal transduction^15^, so the reduced expression of this receptor and its effectors was consistent with diminished peripheral metallic-taste signaling. In parallel, genes supporting taste-cell proliferation and taste-bud/mucosal homeostasis, including Fgf17, Bpifa2, and Adora3, were significantly downregulated (*P* < 0.05) in the cisplatin group. Because Fgf17 is closely related to cell growth and development, its reduction suggests accelerated apoptosis and slower regeneration of taste receptor cells after cisplatin injection^16^. Consistent with this, Ren et al.^12^ reported that cisplatin, even at a lower dose for 14 days, impaired taste function by inhibiting cell proliferation, promoting apoptosis, and delaying taste cell differentiation. Together, these transcriptional changes indicated that cisplatin blunts peripheral taste signaling and disrupts taste-cell turnover, which would reduce the normal aversion to the metallic stimulus.

However, a decrease in taste sensitivity did not fully account for the increased preference for metallic taste (PR > 80%) observed in the TBP test (Fig. 1b). Previous clinical studies showed that chemotherapy patients receiving cisplatin reported a persistent metallic taste in the oral cavity^2,5^. Therefore, one plausible interpretation is that cisplatin induces a sustained “metallic” sensation in mice, making the taste of ferrous sulfate become perceptually familiar and thereby promoting its consumption. In our study, several cytochrome P450 monooxygenase genes—including Cyp4a14, Cyp2c66, Cyp2j5, and Cyp2e1—were significantly upregulated (*P* < 0.05) in the cisplatin group. These genes are known to facilitate the reduction of heme iron from the ferric (Fe³⁺) to ferrous (Fe²⁺) state, thereby increasing intracellular Fe²⁺ levels and rapidly inducing lipid oxidation^17^. Previous studies have also proved that cisplatin treatment might induce excessive reactive oxygen species (ROS) production, which triggers lipid oxidation and subsequently oxidative damage and ferroptosis^18^. Thus, we hypothesize that secondary oxidation products with a metallic odor, such as 1-octen-3-one, could represent one pathway by which cisplatin contributes to an endogenous metallic sensation in mice.

Following LF treatment, genes directly related to taste function, including metallic-taste receptor Tas2r130 and downstream signaling such as α-gustducin (Gnat3) and Trpm5, were significantly upregulated (*P* < 0.05), as confirmed by qPCR (Fig. 4d). LF also markedly downregulated (*P* < 0.05) the expression of Cyp4a14 and Cyp2j5, two genes associated with lipid oxidation. Furthermore, genes related to neurotransmitter transmission, such as Apln and Kcnt1, were significantly upregulated (*P* < 0.05) in the cisplatin group but downregulated (*P* < 0.05) in the LF group, suggesting that LF treatment might reduce signaling pathways associated with bitterness perception and thus alleviate the sensation of endogenous metallic taste^12^. At the same time, LF group showed significantly increased expression (*P* < 0.05) of Chrm3, which is involved in the modulation of intracellular signaling, particularly calcium mobilization^13^. This result indicated that LF supplementation might benefit the recovery of normal neural perception of taste.

Zinc, a cofactor for enzymes involved in taste signal transduction, produced additional transcriptional changes when combined with LF^9,10^. Compared to LF group, co-application of LF and ZnSO_4_ further increased (*P* < 0.05) the expression of genes related to taste function, including Tas2r130 and its downstream signaling (Gnat3, Plcβ2, Trpm5) as well as Tnf (bitter-taste signaling)^19,20^ (Fig. 4d). Furthermore, LF+ZnSO₄ treatment also raised intracellular signaling mediators (Hras, Chrm3) and the DNA-damage sensor H2ax, while decreasing genes governing cell-cycle progression and mitosis (e.g., Nuf2, Mki67, Ndc80, Mad2l1, Kif23, Kif11). Co-application of LF and ZnSO₄ might restrain excessive taste cell proliferation, hence shifting taste cells from a proliferative/stress state toward homeostasis^21^. However, this transcriptional difference did not translate into a measurable behavioral difference between the LF and LF+ZnSO₄ groups (*P* > 0.05). This might be because the behavioral test had reached a performance ceiling with LF alone, potentially masking incremental improvements from the combined treatment. Future studies incorporating targeted neurophysiological approaches will be required to clarify whether the enhanced transcriptional responses induced by LF + ZnSO₄ result in functional improvements at the sensory or neural processing levels.

In the current study, we observed that a large number of genes associated with inflammatory responses were significantly downregulated (*P* < 0.05) in the cisplatin group compared with the control group (Fig. 2d), including Cxcl10, H2-Ab1, Cd74, Cx3cr1, Cd83, Cd209a, Il12b, Pla2g2d, CIITA, H2-Aa, Siglech, and H2-M5. These results suggested a potential impairment in antigen presentation and immune activity in mice. This was consistent with the well-documented immunosuppressive effects of cisplatin, including decreased white blood cell counts, impaired lymphocyte function, and downregulation of immune-related genes and proteins^5,22^. Compared to the saline group, a substantial upregulation of genes correlated with immunity was identified in LF group, including chemokine (Cxcl10), interferon-stimulated antiviral effectors (Ifit1, Irf7, Isg15), antigen-presentation and costimulatory molecules (B2m, Cd209e, Cd3e, Icos), immune homeostasis maintenance (Pla2g2d, Ikzf3, Siglech), and epidermal barrier formation (Lce1β, Lor)^12,23,24^. Thus, at the transcriptional level, LF coordinately restored the expression of immune and epithelial barrier genes that had been suppressed by cisplatin. In addition, co-application of LF and ZnSO₄ was associated with additional upregulation of immune-related genes relative to LF alone, including mucosa-associated genes (Slurp1, Cd74, Cxcl14, Cxcl16, Cxcl17), adaptive-immunity and antigen-presentation genes (Ccl5, Cx3cr1, H2-Aa, H2-Ab1, B2m, Cd4, Ciita)^11,12,24^. These results suggested potential roles in shaping the inflammatory and antimicrobial environment in mucosal/tongue tissues that may enhance mucosal defense and taste cell recovery^12,24^.

In recent years, accumulating evidence has shown that disruption of immune pathways might lead to sustained localized inflammation, which impairs taste cell renewal and taste preferences^1,20^. Taste receptor cells are sensitive to inflammatory insult, and immune dysregulation has been reported to alter taste preference, taste-bud composition, induce apoptosis, and impair the renewal capacity of taste stem cells^12,25,26^. On this basis, we hypothesize that cisplatin-induced suppression of local immune and antigen-presentation pathways contributes to impaired taste-cell renewal. LF, alone or with zinc, supports taste recovery in part by restoring the above pathways. As with the oxidative-stress hypothesis, this immune-mediated mechanism is inferred from mRNA-level changes and requires protein-level and functional confirmation in future work.

Several limitations should be acknowledged in the current study. First, gene expression data do not directly reflect protein abundance or functional activity; future proteomic and immunohistochemical validation would be required to substantiate the proposed mechanisms. Second, taste responses were assessed using a single metallic stimulus against water, without controls for other modalities, and cisplatin might also distort taste coding such that the metallic stimulus is misinterpreted as less aversive. Therefore, electrophysiological recordings, calcium imaging, or histological analyzes of taste buds would help link transcriptional changes to functional outcomes. Third, the treatment experiment enrolled the most severely affected animals, so findings should be interpreted as applying to that subpopulation rather than to all cisplatin-treated mice. Besides, the 48-h TBP test might incorporate post-ingestive feedback, and baseline FeSO₄ avoidance may include non-gustatory contributions such as gastrointestinal effects, altered iron homeostasis, or reduced appetite. Finally, the proposed role of LF in limiting lipid oxidation remains unverified. We therefore encourage future work to directly quantify the lipid-peroxidation products and ROS in vivo to test this hypothesis.

In summary, we found that long-term (3 weeks) exposure of cisplatin in mice induced metallic taste disorder, which might arise from direct and indirect damage to taste function, including decreased expression of genes associated with metallic taste receptor and transduction, accelerated apoptosis of taste cells, and immunosuppression. Both LF and LF+ZnSO₄ supplementation effectively alleviated metallic taste disorder in mice within 14 days. At the transcriptional level, LF significantly upregulated genes associated with metallic taste perception and immune responses, which we propose may support the recovery of taste function. LF might also take effect by inhibiting Fe^2+^-induced lipid oxidation due to its metal-chelating and antioxidant properties. Addition of ZnSO₄ further increased the expression of genes involved in taste signaling, intracellular mediators, and immunity in the oral epithelium. Thus, this study identified potential therapeutic targets for chemotherapy-induced taste disorder and suggests LF and ZnSO₄ as promising food-derived strategies for mitigating chemotherapy-associated taste disturbances.

## Methods

### Animals

Wild-type BALB/c female mice were purchased from Beijing Spelford Biotech. Co., Ltd (Beijing, China). Mice used for experiments were 23–25 days old and 20 to 22 g weight when delivered. Mice were housed at 22 ± 3 °C temperatures and 45 ± 5% relative humidity with a 12/12 h light/dark cycle and free access to standard mice chow and water before two-bottle preference tests. The procedures of animal handling were performed according to the Guidelines for the Care and Use of Experimental Animals and were approved by the Veterinary Ethics Committee of Chengdu University (Approval No. EAEC-31219). The mice were acclimated to the new environment for 1 week before the two-bottle preference test.

### Two-bottle preference (TBP) test

**Fig. 5:**
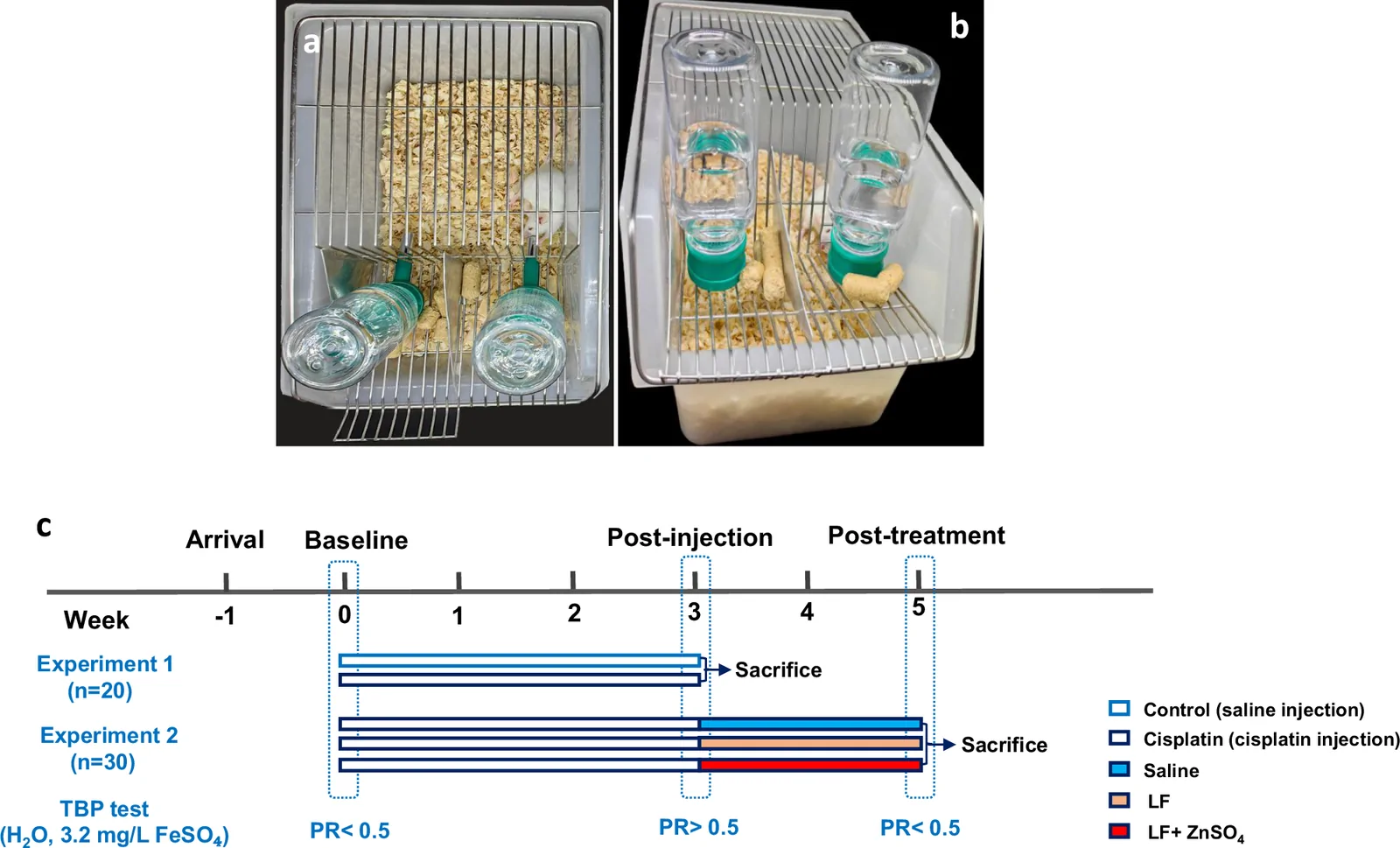
Experimental apparatus and study design for the two-bottle preference test. Top view (**a**) and front view (**b**) of the two-bottle preference test apparatus; schematic representation of the experimental layout for experiments 1 and 2 showing groups and the time of behavioral testing (**c**).

Two-bottle preference (TBP) test (48 h) was performed following the procedure developed in previous studies^27^. Each mouse cage was equipped with two water bottles (120 mL each) on each side (Fig. 5). The bottles were designed to release fluid only when the mice actively engaged in sucking behavior, and remained leak-proof at all other times regardless of placement. Mice were housed individually 48 h before starting the TBP test to adapt to the environment. During this time, the animals were provided with deionized water (Wahaha, Hangzhou, China) in both of the bottles. For the metallic taste TBP test, one bottle was an FeSO_4_ (food-grade, Yisheng Co., Ltd, Shenzhen, China) solution prepared daily with deionized water, and the other one was deionized water. The positions of the two bottles were switched every 24 h to avoid lateral preference, and the total fluid intake was recorded every 24 h. Daily food intake was measured by providing each mouse with a fixed amount of food (5 g/day), weighing the remainder the following day, and calculating the difference. Measurements were taken at the same time each day throughout the experimental period. Throughout the TBP testing period, mice were given free access to food and fluid. Metallic taste preference ratio was calculated according to the following formula:

$${\rm{Metallic}}\; {\rm{taste}}\; {\rm{preference}}=\frac{48{{\rm{h}}\; {\rm{FeSO}}}4{\rm{solution}}\; {\rm{intake}}({\rm{g}})}{48{\rm{h}}\; {\rm{FeSO}}4{\rm{solution}}\; {\rm{intake}}\left({\rm{g}}\right)+\,48{\rm{h}}\; {\rm{deionized}}\; {\rm{water}}\; {\rm{intake}}\left({\rm{g}}\right)}\times 100$$

### Experimental design

#### Experiment 1

Female BALB/c mice (*n* = 30) were purchased and screened for metallic taste sensitivity by TBP test at a concentration of 3.2 mg/L FeSO₄ solution. Of these, 23 mice met the inclusion criterion of a baseline metallic-taste PR < 50%, from which 20 mice were selected on the basis of balanced body weight for the following tests. This was the initial stage of the experiment and labeled as baseline (week 0). Then the mice were randomly divided into two groups of 10 mice each with balanced weight: (1) control group (saline); (2) cisplatin group (cisplatin, 3.5 mg/kg). We administered cisplatin (Cis, 3.5 mg/kg) to mice at dosages within the range used for cancer treatment to induce metallic taste abnormality in mice^11^. For both groups, one dose of cisplatin/ saline with the same amount (0.1 mL/10 g) was injected intraperitoneally two times a week (day 1 and day 4) for a total of 3 weeks. Then the metallic taste PR of mice was evaluated by TBP test (deionized water, 3.2 mg/L FeSO₄ solution) and this time point was recorded as the post-injection stage (week 3). At the end of experiment 1, all mice were sacrificed by euthanasia and tongue, thymus and spleen tissues were surgically excised.

#### Experiment 2

Female BALB/c mice (*n* = 40) were purchased and screened for metallic taste sensitivity as described above. Of these, 34 mice met the inclusion criterion of a baseline metallic-taste PR < 50%. All eligible mice were injected with cisplatin at 3.5 mg/kg twice a week (day 1 and day 4) for 3 weeks to induce metallic taste abnormality. The metallic taste PR was then evaluated by TBP test (deionized water, 3.2 mg/L FeSO₄ solution) at the post-injection stage (week 3). The 30 mice with the highest PR, indicating the most pronounced metallic taste disorder, were selected for the subsequent treatment phase. The mice (*n* = 30) were randomly divided into three groups of 10 mice each: (1) saline group (control); (2) LF group (lactoferrin in a dose of 120 mg/kg); (3) LF+ ZnSO₄ group (120 mg LF/kg and 27.5 mg ZnSO₄/kg). Lactoferrin was dissolved in saline at a concentration of 12 mg/mL to achieve a dose of 120 mg/kg. Zinc sulfate was dissolved at a concentration of 2.75 mg/mL to achieve a dose of 27.5 mg/kg. In the LF+ZnSO₄ group, both compounds were co-administered in the same gavage solution at the above concentrations. The dosage of LF was determined by converting the human dose to a mouse-equivalent dose, according to our previous study^5^, using allometric scaling based on body surface area as recommended by the U.S. FDA guidelines. The dosage of zinc was determined according to the protocol established by Ahn et al.^28^. Each group was administered by oral gavage at a volume of 10 mL/kg once daily for 14 consecutive days. During this period, mice were given free access to food and water. Then the metallic taste PR of mice was evaluated by TBP test (deionized water, 3.2 mg/L FeSO₄ solution), and this time point was recorded as the post-treatment stage (week 5). The workflow was illustrated in Fig. 5c. At the end of experiment 2, all mice were sacrificed by euthanasia and tongue, thymus and spleen tissues were surgically excised.

### Thymus and spleen index

The thymus and spleen were rinsed briefly with ice-cold saline to remove residual blood and then blotted dry with absorbent paper and accurately weighed to calculate the organ coefficient. This calculation is performed using the following formula: organ coefficient (mg/g) = organ weight (mg) / body weight (g).

### RNA extraction and RNA-Seq analysis

Total RNA was extracted from the tongue using the HiPure Universal RNA Mini Kit and RNA concentration was measured using a Qubit 4.0 Fluorometer and Nanodrop One. RNA-Seq analysis was conducted using an Illumina sequencing system, and the transcription levels of the genes were assessed using the Fragments Per Kilobase per Million fragments (FPKM) method calculated by DESeq2 software. Genes between two groups with FDR ≤ 0.05 and |log_2_(fold change)| ≥ 1.5 were identified as differentially expressed genes (DEGs). DEGs were functionally classified by Gene Ontology enrichment analysis (GO, http://www.geneontology .org/). Protein-protein interaction (PPI) analysis of DEGs was performed using the STRING database (http://string-db.org/) with a confidence threshold of 0.4. The resulting PPI network was imported into Cytoscape (v3.10.2) to construct the network diagram. Degree centrality (degree) was calculated using the “cytohubba” plug-in to screen for the top hub genes.

### Quantitative real-time PCR

First-strand cDNA was synthesized using a PrimeScript RT Master Mix (catalog #RR036A, Takara) and primers used in this study were synthesized by Tsingke Biotechnology Co., Ltd (Beijing, China). Quantitative real-time PCR was performed on an Analytik Jena Real-Time PCR System with 10 μL volumes including 5 μL 2×SYBR Green SuperMix, 0.5 μL of each primer (F and R, 0.2 mM), 1 μL of DNA template, and 1 μL of double distilled H_2_O. Amplification conditions were: initial denaturation at 95 °C for 3 min, followed by 39 cycles for 10 s at 95 °C, 30 s at 60 °C, and a final extension of 5 min at 72 °C. The relative expression levels were calculated using the 2^-ΔΔCt^ method. Statistical analyzes of qPCR data were performed using Student’s *t*-test (experiment 1) and one-way ANOVA (experiment 2). To account for multiple comparisons across the validated genes, *P* values were adjusted using the false discovery rate (FDR) correction, with adjusted *q* < 0.05 considered statistically significant. Primer sequences used were listed in Supplementary materials (Table S1).

### Statistical analysis

Statistical analyzes were conducted using SPSS version 16.0 and GraphPad Prism 9.5 software (GraphPad Software, La Jolla, CA). Data were expressed as mean ± standard deviation, and statistical significance was determined by Student’s *t*-test and one-way analysis of variance (ANOVA) with Dunnett’s multiple-comparisons test. A *P*-value of <0.05 was considered statistically significant.

## Supplementary information

Supplementary materials

## Data Availability

The data that support the fi ndings of this study are available from the corresponding author, Aili Wang, upon reasonable request.
