# Supplementary material for: Lactoferrin and zinc sulfate supplementation alleviate cisplatin-induced taste disorder: underlying mechanisms via taste and immune modulation
Source: NPJ Sci Food. 2026 Aug 27;10:281. doi: 10.1038/s41538-026-01096-9 (PMC13574781; doi:10.1038/s41538-026-01096-9)
Supplement: Supplementary file 1 [file 41538_2026_1096_MOESM1_ESM.pdf]

Table S1. Primer sequences for quantitative PCR.

Gene	Forward primer	Reverse primer
Tas2r130	TTCTCATAGCCACCTCCAG	TACAAAGCACCCTCACAGAT
Gnat3	GTGCCTATGACATGGTGCTTG	CAGAACAATGGAGGTGGTTGC
TRPM5	CAGGAAGGAAGCCCAGCATAA	AAGTCCACTCTGTGTGCCGT
PLCβ2	GGTGGAAGAGGAGGAAGA	ATTGACAAGGCTGGACATT
H2-Ab1	TGTCGTCATCTCCCTGTC	CTTGGCTGGGTAGAAATCTG
Cyp4a14	GAGACCCTGGAAGTTGTGG	GGTTCTTTGCGGTAACAGA
CD74	GCAGTGGCTCTTGTTTGA	TGTGGCTGACTTCTTCCT
Lce1β	GGAAGGGCACCATGACCTT	GCTCAAGGATGAGTTGGTTG
Tnf	AGCCCCCAGTCTGTATCCTT	CTCCCTTTGCAGAACTCAGG
Cx3cr1	CACTGAAGTGCCAGCCATTC	GAGGGCAAGAGGAAAACTCC
Chrm3	GTGCATAATCGGTTCCTGGA	AGGTAGACCAGGTTGATGCC
Fgf17	AGCGTGGAAGAAGTTCAGGA	AAACAATTCAACTGCAGCCA

Sex differences in metallic taste sensitivity test

In order to investigate the effect of sex on metallic taste sensitivity in mice, male (n=10) and female (n=10) mice were performed the metallic taste TBP test using a series of concentrations of $FeSO_4$ solutions in ascending order, including 0, 0.8, 1.6, 3.2, and 6.4 mg/L. Between the two concentrations, there was a 48-hour wash-out period: the mice maintained the access to both bottles and each containing deionized water. The weight of mice was recorded every 48 h. When metallic taste preference ratio (PR) greater than 0.5, it indicated that the mice showed a preference for $FeSO_4$ solution, whereas a PR less than 0.5 reflected an aversion to metallic taste. The greater the absolute difference between the PR value and 0.5, the stronger the mice's preference or aversion toward the metallic taste.[1]

We firstly assessed the sex differences in metallic taste sensitivity in mice through metallic taste TBP test. In the preliminary test, we assessed the preference ratios (PR) for metallic taste in both female and male mice using a series of $FeSO_4$ solutions including 0, 0.1, 0.2, 0.3, 0.4, 0.5, 0.6, and 0.8 mg/L. However, we observed an adaptation effect in both sexes: mice exhibited higher metallic taste PR at higher $FeSO_4$ concentrations, suggesting a reduced aversive response to the metallic taste. This adaptation may have masked the true sensitivity to metallic stimuli at higher concentrations, thus we increased the concentration gradient of $FeSO_4$ in the formal experiments.

As shown in Fig. S1a, female mice exhibited apparent aversive response to $FeSO_4$ solution starting at a concentration of 0.8 mg/L. Notably, more than half of the female mice demonstrated a metallic taste PR below 10 %, and this aversion tendency became more significant along with the increase of $FeSO_4$ concentrations. When the concentration of

$FeSO_4$ reached 6.4 mg/L, the preference ratio of all female mice were lower than 50 %. In contrast, while male mice also displayed an aversion to $FeSO_4$ solution at 0.8 mg/L, this tendency diminished according to the increase of $FeSO_4$ concentrations. When the concentration of $FeSO_4$ achieved 6.4 mg/L, the preference ratio of more than half of the male mice were higher than 50 %. This result suggested that male mice exhibited stronger taste adaptation to the metallic taste induced by ferrous ions. Our result was consistent with a previous study that male mice accepted a quinine diet (bitter diet) much faster than females.[2]

The metallic taste PR of female mice were significantly lower ($P < 0.05$) than that of male mice at the concentration of 3.2 mg/L ($P < 0.05$) and 6.4 mg/L ($P < 0.01$), indicating a higher taste discrimination at these concentrations. Furthermore, the variation of PR of female mice at 3.2 mg/L and 6.4 mg/L was much smaller than the previous $FeSO_4$ concentrations as well as male PR. Sex-based differences in bitterness sensitivity have been reported in mice. Studies have shown that female mice often exhibit higher sensitivity to certain bitter compounds compared to males, as reflected by lower PR or increased aversive responses in behavioral taste tests.[3] This difference may be attributed to hormonal regulation, genetic factors, or variations in taste receptor expression between sexes.[2,3] Although male and female mice showed different sensitivities to $FeSO_4$, there were no significant changes ($P < 0.05$) in total water intake or body weight, indicating that healthy mice did not reject food or water due to the perception of metallic taste (Fig. S1b and S1c). Thus, female mice were selected for subsequent experiments and 3.2 mg/L of $FeSO_4$ solution was chosen as the taste threshold for metallic taste screening in mice.

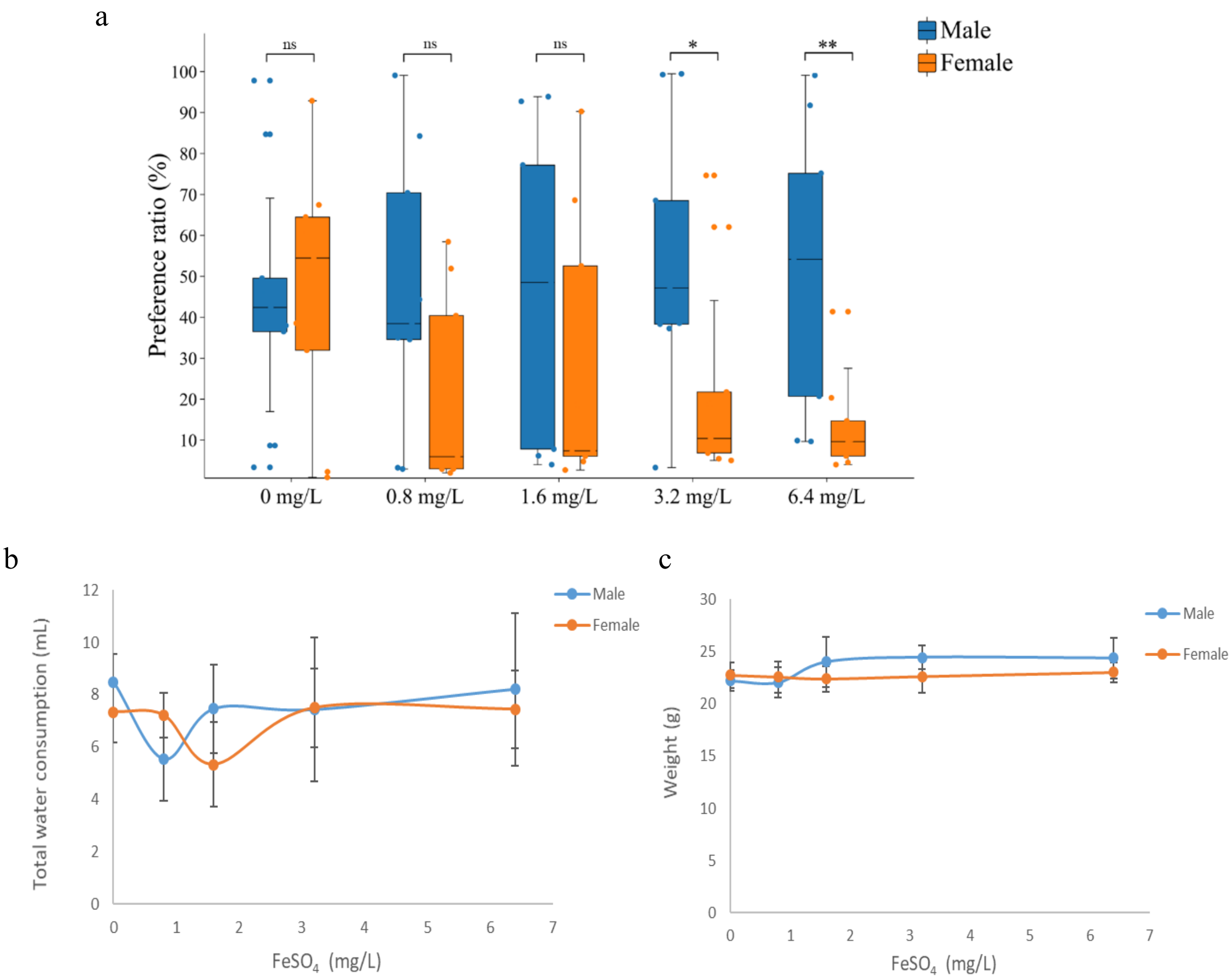

Figure S1. Metallic taste preference ratios (a), total water consumption (b), and weight (c) of male and female mice in TBP test using deionized water and different concentrations of $FeSO_4$ solution including 0, 0.8, 1.6, 3.2, and 6.4 mg/L. All data were presented as means ± standard deviation. Statistical significance was conducted by unpaired t-test and represented by asterisks: $^{**}P < 0.01$, $^{*}P < 0.05$. Data points in box plots indicated individual animal.

Reference

(1) Verharen, J. P., de Jong, J. W., Zhu, Y., & Lammel, S. A computational analysis of mouse behavior in the sucrose preference test. *Nat. Commun.* **14(1)**, 2419 (2023).

(2) Beede-James, K. F., Gutierrez, V. A., Brooker, S. L., Martin, L. E., & Torregrossa, A. M. Sex differences in diet-mediated salivary protein upregulation. *Appetite.* Article 107888 (2025).

(3) Clarke, S. N., & Ossenkopp, K. P. Taste reactivity responses in rats: influence of sex and the estrous cycle. *Am. J. Physiol. Regul. Integr. Comp. Physiol.* **274(3)**, 718-724 (1998).
